# Role of ^18^F-FDG-PET in renal tumors: insights from WHO 2022 classification

**DOI:** 10.1007/s11604-025-01761-1

**Published:** 2025-04-07

**Authors:** Kohei Hirota, Yoshiko Ueno, Munenobu Nogami, Toshiki Hyodo, Takahiro Tsuboyama, Keitaro Sofue, Naoya Ebisu, Takuto Hara, Izumi Imaoka, Takamichi Murakami

**Affiliations:** 1 Department of Radiology, Kobe University Graduate School of Medicine, 7-5-2, Kusunoki-cho, Chuo-ku, Kobe, Hyogo 650-0017 Japan; 2 Division of Medical Imaging, Biomedical Imaging Research Center, University of Fukui, 23-3, Matsuoka Shimoaizuki, Yoshida Gun Eiheiji Cho, Fukui, Fukui 910-1104 Japan; 3 Department of Pathology, Kobe University Graduate School of Medicine, 7-5-2, Kusunoki-cho, Chuo-ku, Kobe, Hyogo 650-0017 Japan; 4 Department of Urology, Kobe University Graduate School of Medicine, 7-5-2, Kusunoki-cho, Chuo-ku, Kobe, Hyogo 650-0017 Japan

**Keywords:** FDG-PET, Renal tumor, WHO 2022 classification

## Abstract

The objective of this article is to provide a comprehensive overview of the imaging characteristics of various renal cell tumors using 18F-fluorodeoxyglucose (FDG)-positron emission tomography (PET), based on the latest WHO-2022 classification. Due to the physiological accumulation of FDG in the kidneys, the clinical utility of FDG-PET in the evaluation of renal tumors has traditionally been considered limited. However, recent studies have re-evaluated its potential value. FDG-PET has demonstrated particular utility in detecting metastases and postoperative recurrence of renal cell carcinoma (RCC), as well as in identifying RCC in patients with chronic kidney failure, where FDG excretion into the urinary tract is reduced. Renal tumors are occasionally detected incidentally on FDG-PET, and FDG uptake varies depending on the tumor subtype. Therefore, a comprehensive understanding of these imaging characteristics is clinically important, as it may serve as a valuable guide for subsequent diagnostic evaluations. Furthermore, recent advancements in the development of novel PET tracers hold promise for future applications in the imaging of renal tumors. We believe that the insights gained from this study will contribute to routine diagnostic practice and the planning of future research.

## Introduction

**Fig. 1:**
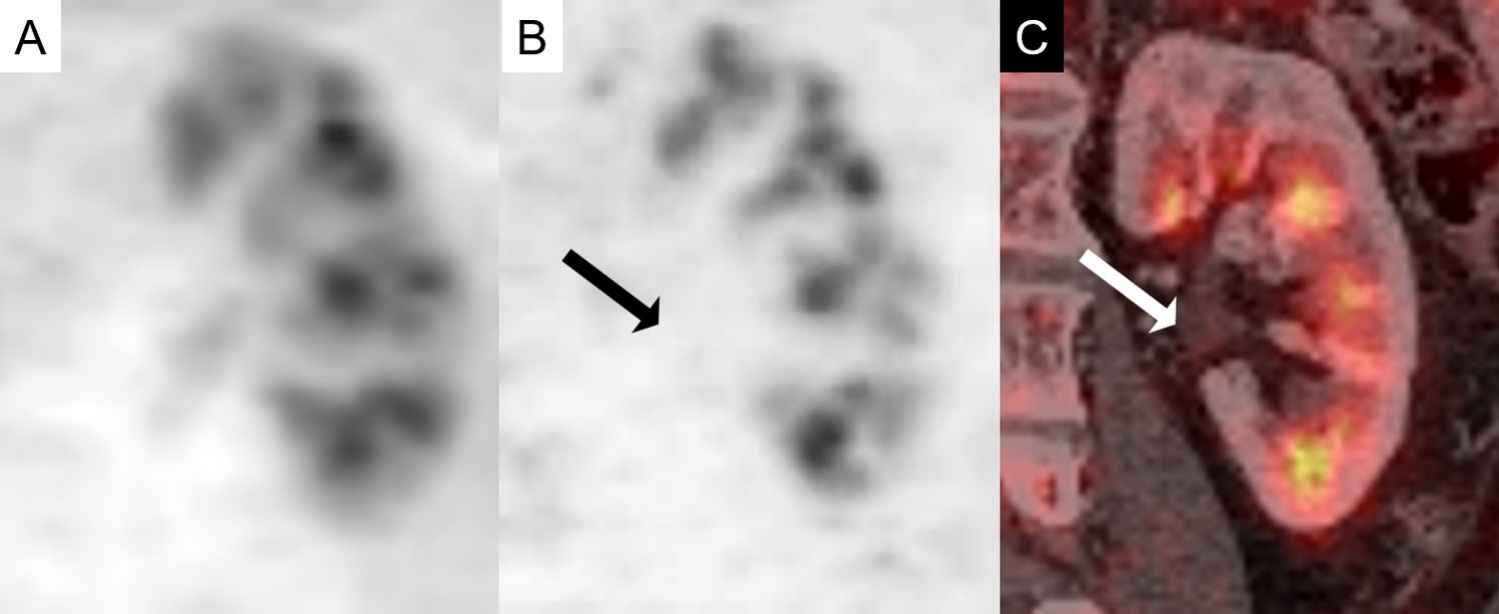
Physiological ^18^F-fluorodeoxyglucose (FDG) uptake of the kidneys. **A** Coronal FDG-positron emission tomography (PET) shows higher physiological accumulation in the renal medulla than in the cortex. **B** Coronal FDG-PET and **C** its fused image with contrast enhanced CT after administration of diuretics. Urinary FDG concentrations excreted in the renal pelvis (arrows) are decreased, as is accumulation in the renal cortex. Accumulation in the renal medulla is still present

^18^F-Fluorodeoxyglucose (FDG)-positron emission tomography (PET) is widely employed in the management of malignant tumors. However, physiological excretion of FDG through the kidneys reduces contrast between normal renal tissue and pathological lesions, making it generally considered less effective for the evaluation of renal tumors. FDG uptake predominantly occurs in the renal medulla, where it persists even after diuretic administration (Fig. 1), with lower uptake in the cortex. Due to this physiological accumulation, FDG-PET is generally not used for diagnosing renal tumors; however, incidental detection of renal masses during FDG-PET is common. Since FDG reflects tumor glucose metabolism, recognizing physiological accumulations and assessing FDG uptake in renal tumors can aid in diagnosis. Furthermore, recent studies have suggested that FDG-PET is useful for detecting metastases or postoperative recurrence of renal cell carcinoma (RCC), and RCC arising in acquired cystic disease (ACD) in dialysis patients. In 2022, the World Health Organization (WHO) revised the classification of renal cell carcinoma (WHO-2022 classification), including the broad categorization into two main groups, morphologically defined renal tumor and molecularly defined renal carcinoma, and the elimination of the subclassification for papillary renal cell carcinoma [1] (Table 1). This paper reviews the FDG-PET and histopathological findings of renal cell tumors confirmed at our institution, in conjunction with existing reports, based on the WHO-2022 classification.

**Table 1:** The 2022 World Health Organization classification of the renal cell tumours [1]

Renal cell tumours
*-Clear cell renal tumours* Clear cell renal cell carcinoma Multilocular cystic renal neoplasm of low malignant potential *-Papillary renal tumours* Papillary adenoma Papillary renal cell carcinoma *-Oncocytic and chromophobe renal tumours* Oncocytoma Chromophobe cell renal carcinoma Other oncocytic tumours of the kidney *-Collecting duct tumours* Collecting duct carcinoma *-Other renal tumours* Clear cell papillary renal cell tumour Mucinous tubular and spindle cell carcinoma Tubulocystic renal cell carcinoma Acquired cystic disease–associated renal cell carcinoma Eosinophilic solid and cystic renal cell carcinoma Renal cell carcinoma, NOS *-Molecularly defined renal carcinomas* TFE3-rearranged renal cell carcinomas TFEB-altered renal cell carcinomas ELOC (formerly TCEB1)-mutated renal cell carcinoma Fumarate hydratase-deficient renal cell carcinoma Hereditary leiomyomatosis and renal cell carcinoma syndrome–associated renal cell carcinoma Succinate dehydrogenase-deficient renal cell carcinoma ALK-rearranged renal cell carcinomas Medullary carcinoma, NOS SMARCB1-deficient medullary-like renal cell carcinoma SMARCB1-deficient undifferentiated renal cell carcinoma, NOS SMARCB1-deficient dedifferentiated renal cell carcinomas of other specific subtypes

### Abstract of changes in WHO-2022 classification

In WHO-2022 classification, renal tumors are classified as “morphologically defined” or “molecularly defined.” Morphologically defined tumors rely on histopathology, while molecularly defined ones need specific molecular findings. Three new tumor types were added, with some renaming and redefinitions. The two-tier classification of papillary renal cell carcinoma was removed, and oncocytic and chromophobe renal tumors were redefined (Table 1) [1].

### Patient selection

Clinically indicated cases that underwent PET/CT or PET/MRI examinations at Kobe University Hospital between March 2011 and May 2023, and for which a pathological diagnosis of renal tumors had been established, were identified via a search of the electronic medical records and imaging database. For cases prior to 2022, an experienced urologic pathologist (TH, with 8 years of experience) re-examined the specimens and re-diagnosed them according to the WHO-2022 classification. Two radiologists (YU, with 16 years of experience, and KH, with 3 years of experience) then evaluated the imaging and pathological findings in consensus to select the cases. Ultimately, the nine cases described below were included in this pictorial essay. This pictorial essay was approved by the Institutional Review Board of Kobe University Hospital, and the requirement for providing explanations to patients and obtaining their informed consent was waived.

### FDG-PET CT/MRI

The cases presented in this article were imaged using the following devices: PET/MRI (Signa PET/MR, GE Healthcare, USA) and PET/CT (Discovery 690, Discovery MI Gen2, GE Healthcare, USA). The detailed imaging protocols are provided in Table 2.

**Table 2:** PET scan parameters

Scanner	Signa PET/MR	Discovery 690	Discovery MI Gen2
Corresponding figure #	2, 10	3, 4, 5, 6, 7, 8	9
Photomultiplier	SiPM	Analog	SiPM
Crystal	LYSO	LYSO	LYSO
Administrated [^18^F]FDG	3.5 MBq/kg	3.5 MBq/kg	3.5 MBq/kg
Matrix	256 × 256	192 × 192	256 × 256
Emission scan duration	2.5 min/bed	2 min/bed	2.5 min/bed
Reconstruction	TOF-BSREM	TOF-OSEM	TOF-BSREM
Subsets	NA	28	NA
Iterations	NA	2	NA
Filter	NA	Gaussian 4mm	NA
β value	550	NA	450
Point Spread Function	On	On	On
Respiratory gating	On	Off	On

*PET* positron emission tomography, *SiPM* silicon photomultiplier, *LYSO* lutetium-yttrium-orthosilicate, *FDG* fluorodeoxyglucose, *TOF* time of flight, *BSREM* block sequential regularized expectation maximization, *OSEM* ordered subset expectation maximization, *NA* not applicable

### Clear cell renal cell carcinoma (ccRCC)

**Fig. 2:**
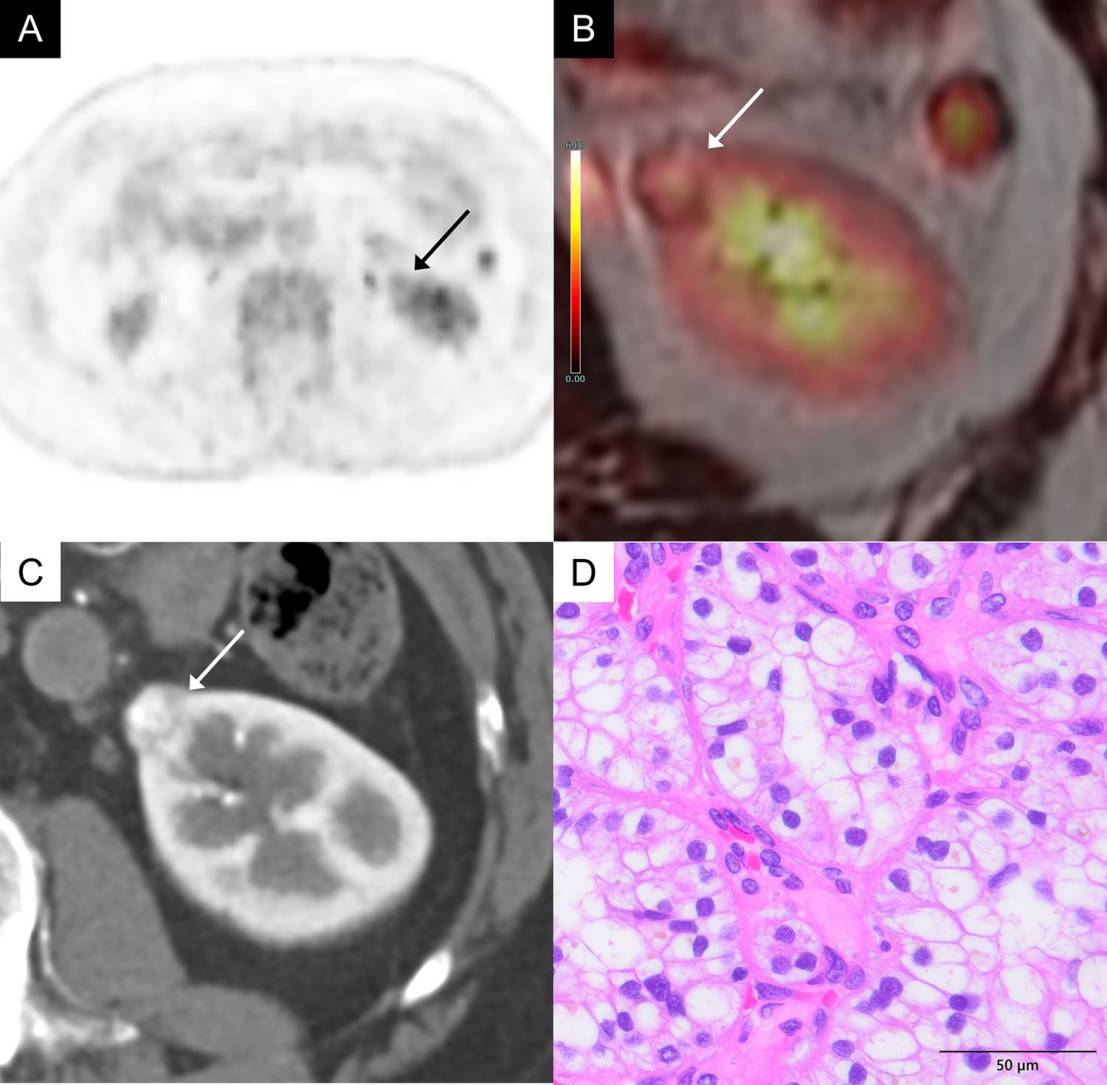
A 60-year-old man with a clear cell renal cell carcinoma (World Health Organizaion/International Society of Urological Pathology (WHO/ISUP) grade 2) of the left kidney.** A** In the FDG-PET image (grayscale), the uptake of the left renal tumor (arrow) is almost the same as that of the renal cortex, making tumor identification difficult. **B** Axial FDG-PET/magnetic resonance imaging (MRI) image shows mild FDG uptake in a mass of the left kidney (arrow), showing a maximum standardized uptake value (SUVmax) 2.8, while normal renal cortex shows an SUVmax 2.3. **C** Axial contrast-enhanced computed tomography (CT) in the corticomedullary phase shows a heterogeneously intense enhancement in the mass (arrow). **D** The tumor cells have predominantly clear cytoplasm. Conspicuous nucleoli are visible only at a high-power view (× 400)

**Fig. 3:**
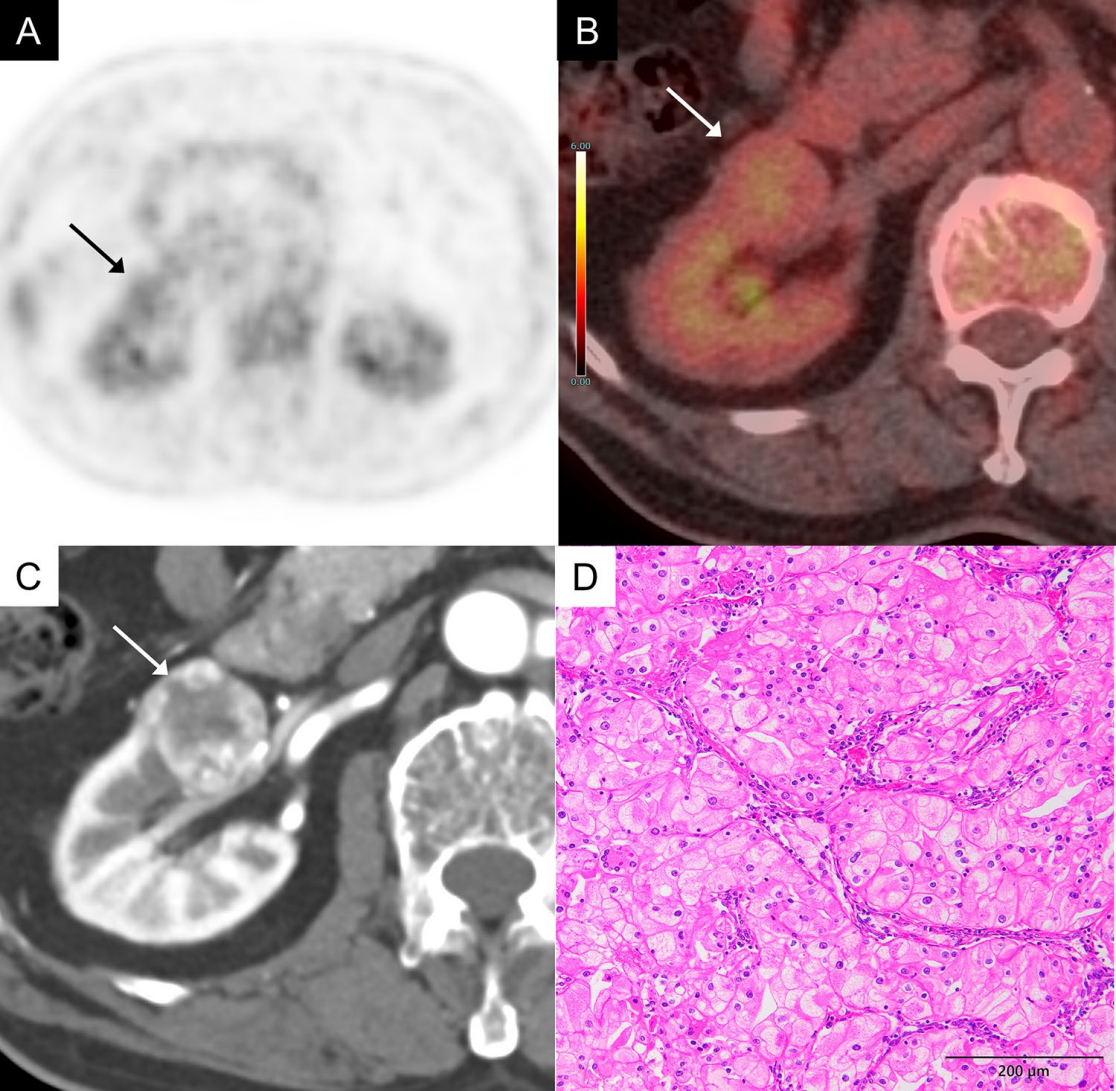
A 79-year-old man with a clear cell renal cell carcinoma (WHO/ISUP grade 3) of the right kidney. **A** Axial FDG-PET image (gray scale) shows mild FDG uptake in a mass of the right kidney (arrow). **B** Axial FDG-PET/CT image shows mild FDG uptake in a mass of the right kidney (arrow), showing an SUVmax 2.9, while normal renal cortex shows an SUVmax 2.3. **C** Axial contrast-enhanced CT in the corticomedullary phase shows a heterogeneously intense enhancement in the mass (arrow). **D** The tumor cells have distinct, eosinophilic nucleoli and clear cytoplasm. These nucleoli are visible at low magnification (× 100)

CcRCC is the most common malignant renal tumor. CcRCC generally shows FDG uptake comparable to or higher than renal cortex (Figs. 2, 3) [2, 3]. In well-differentiated ccRCC, however, high glucose-6-phosphatase activity may reduce FDG retention, resulting in lower uptake. The WHO/International Society of Urological Pathology (ISUP) grading system is widely used to assess nuclear atypia in ccRCC, and studies have shown a correlation between higher nuclear atypia grades and higher maximum standardized uptake value (SUVmax) on FDG-PET. A retrospective analysis of 125 ccRCC cases revealed that high-grade tumors (grade 3 or 4) had significantly higher SUVmax than low-grade tumors (grade 1 or 2) (median SUVmax: 7.3 vs. 3.1, *P* < 0.001) [4].

### Papillary renal cell carcinoma (papRCC)

**Fig. 4:**
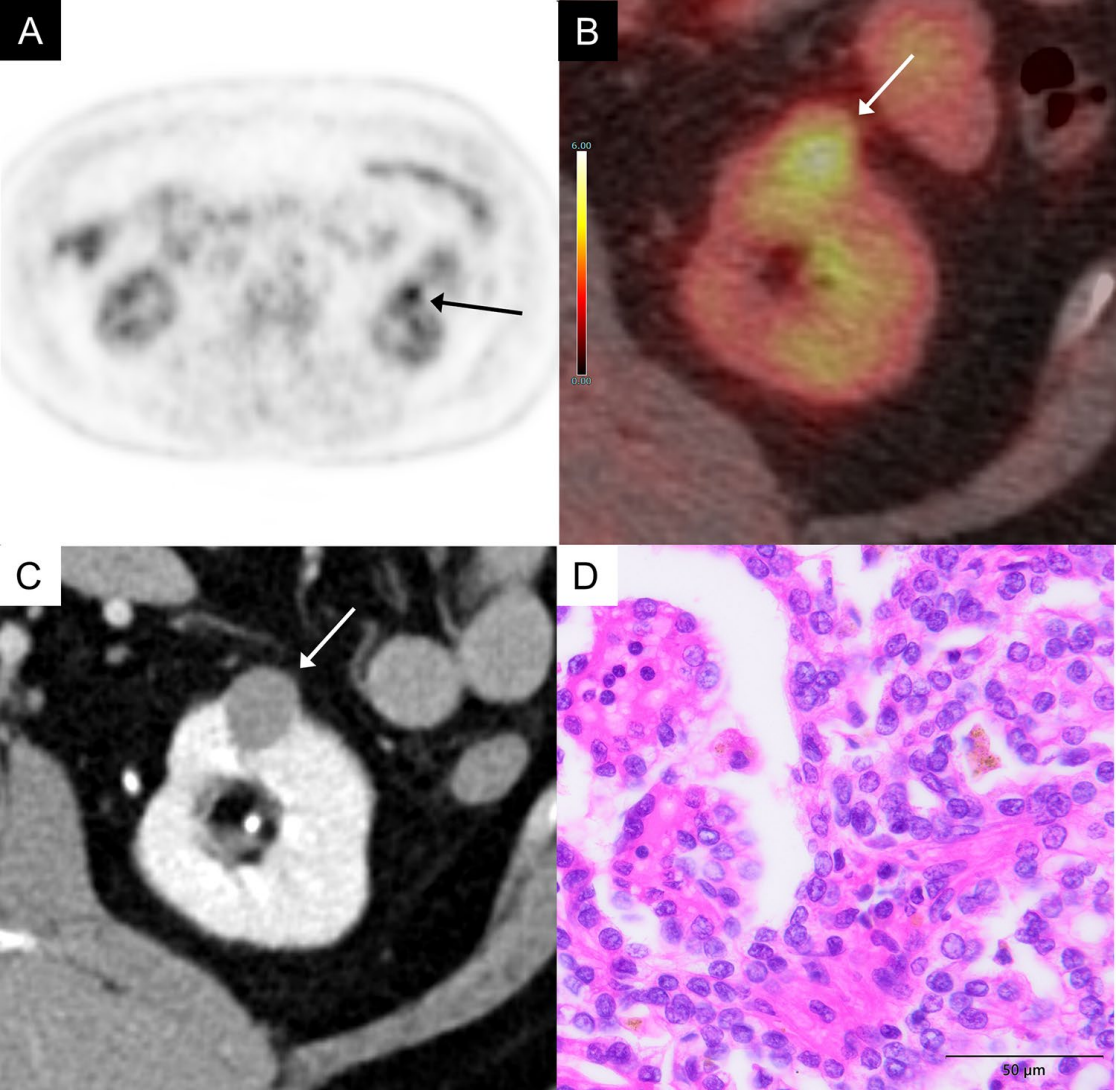
A 45-year-old man with a papillary renal cell carcinoma (WHO/ISUP grade 2) of the left kidney. **A** Axial FDG-PET image (gray scale) shows moderate FDG uptake in a mass of the left kidney (arrow). **B** Axial FDG-PET/CT image shows moderate FDG uptake in a mass of the left kidney (arrow), showing an SUVmax 5.4, while normal renal cortex shows an SUVmax 2.8. **C** Axial contrast-enhanced CT in the nephrographic phase shows a poor enhancement in the mass (arrow). **D** The tumor exhibits predominantly tubular and cribriform structures, with a papillary pattern in several regions. Conspicuous and eosinophilic nucleoli are visible only at a high-power view (× 400)

PapRCC is the second most common malignant renal tumor, following ccRCC. Similar to ccRCC, papRCC generally shows FDG uptake comparable to or higher than renal cortex (Fig. 4) [2, 3]. In an FDG-PET analysis of 38 papRCC cases, 81.5% showed elevated FDG uptake, with high-grade tumors (grade 3 or 4) exhibiting significantly higher SUVmax compared to low-grade tumors (grade 1 or 2) (mean SUVmax: 9.44 vs. 4.83, *P* = 0.008) [5].

### Chromophobe renal cell carcinoma (chRCC) and oncocytoma

**Fig. 5:**
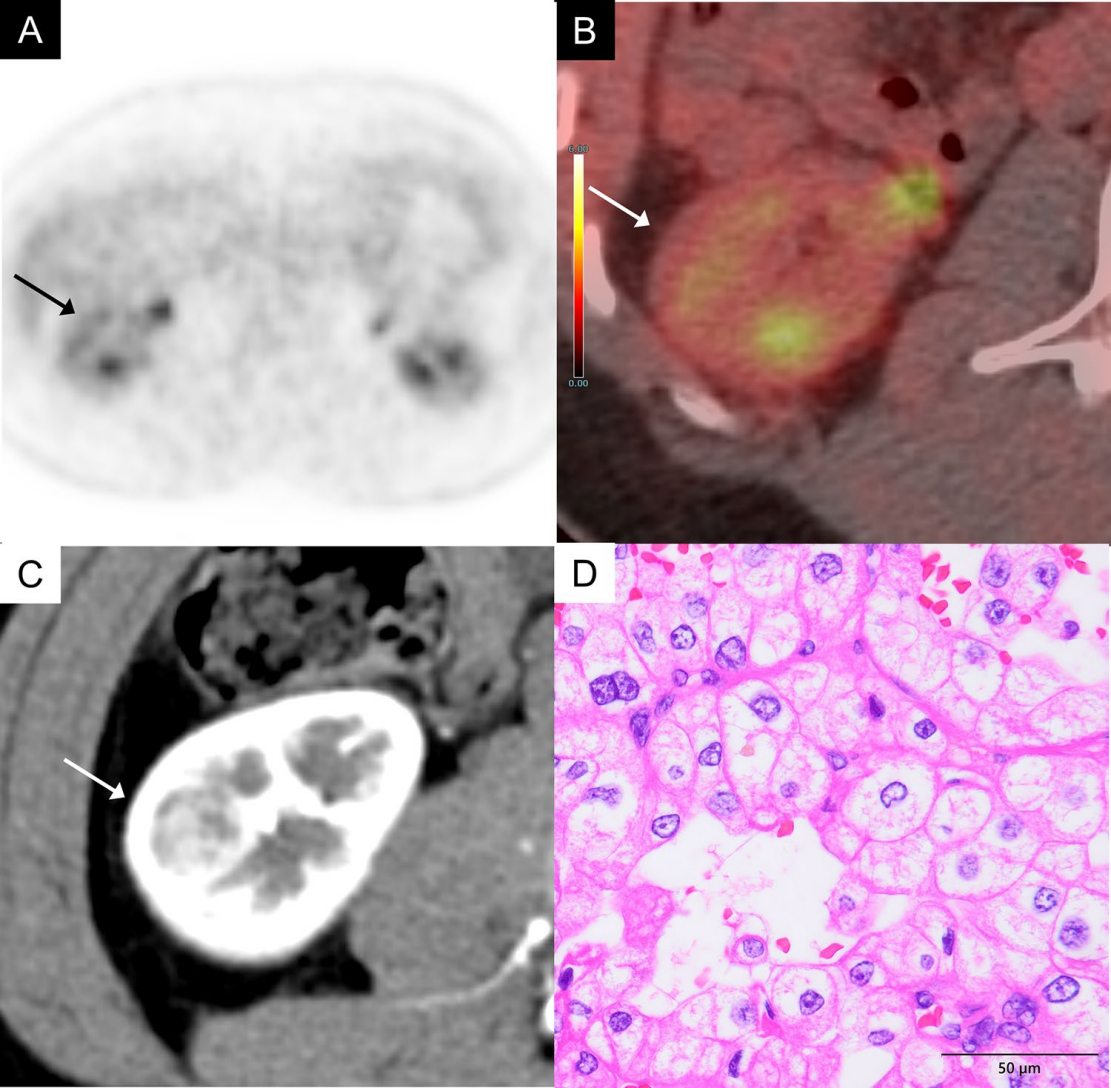
A 35-year-old man with a chromophobe renal cell carcinoma (WHO/ISUP grade 3) of the right kidney. **A** In the FDG-PET image (grayscale), the uptake of the left renal tumor (arrow) is almost the same as that of the renal cortex, making tumor identification difficult. **B** Axial FDG-PET/CT image shows mild FDG uptake in a mass of the right kidney (arrow), showing an SUVmax 2.7, while normal renal cortex shows an SUVmax 2.1. **C** Axial contrast-enhanced CT in the corticomedullary phase shows a heterogeneous enhancement in the mass (arrow). **D** The tumor cells have wrinkling nuclear membranes with eosinophilic cytoplasm. Some tumor cells have perinuclear halo or binuclear

ChRCC is a relatively rare malignant renal tumor compared to ccRCC and papRCC. It tends to exhibit lower FDG uptake than these other histological types (Fig. 5), with mean SUVmax 1.8 [3]. Oncocytoma, which is classified within the same group of “oncocytic and chromophobe renal tumors” as chRCC, often exhibit low FDG uptake like chRCC. However, there have been reports of oncocytomas showing high FDG accumulation [6], indicating a diverse range of imaging findings. It remains a challenging task to differentiate between chRCC and oncocytoma based solely on imaging findings, as they can coexist pathologically.

### Collecting duct carcinoma

**Fig. 6:**
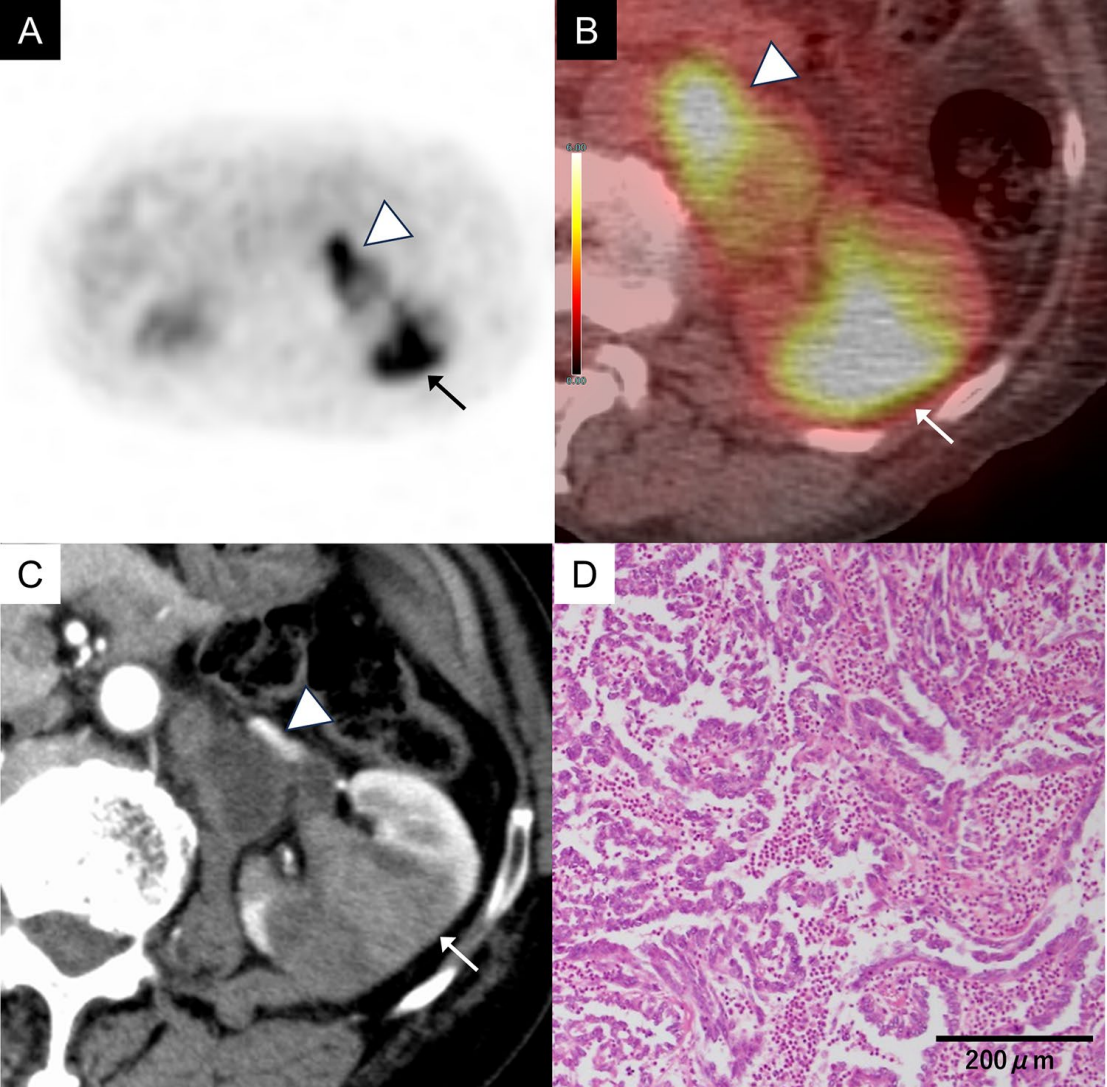
A 77-year-old woman with a collecting duct carcinoma of the left kidney. **A** Axial FDG-PET image (grayscale) shows moderate FDG uptake in left kidney (arrow) and hilar lymph nodes of the left kidney (arrowhead). **B** Axial FDG-PET/CT image shows moderate FDG uptake in a mass of the left kidney (arrow), showing an SUVmax 7.8, while normal renal cortex shows an SUVmax 2.2. FDG uptake is also increased in hilar lymph nodes of the left kidney (arrowhead), suggesting a metastasis of the tumor of the left kidney. **C** Axial contrast-enhanced CT in the corticomedullary phase shows a poor enhancement in the mass (arrow) and the enlarged hilar lymph nodes of the left kidney (arrowhead). **D** Tumor cells are forming papillary and glandular structures

Collecting duct carcinoma is a rare and highly aggressive renal tumor that demonstrates elevated FDG uptake. One study revealed that collecting duct carcinoma showed FDG uptake higher than the renal cortex, with a mean SUVmax of 6.27 [7], and in our case, FDG uptake showed an SUVmax of 7.8, yielding similar results (Fig. 6).

### Other renal tumors

#### Acquired cystic disease-associated renal cell carcinoma (ACD-RCC)

**Fig. 7:**
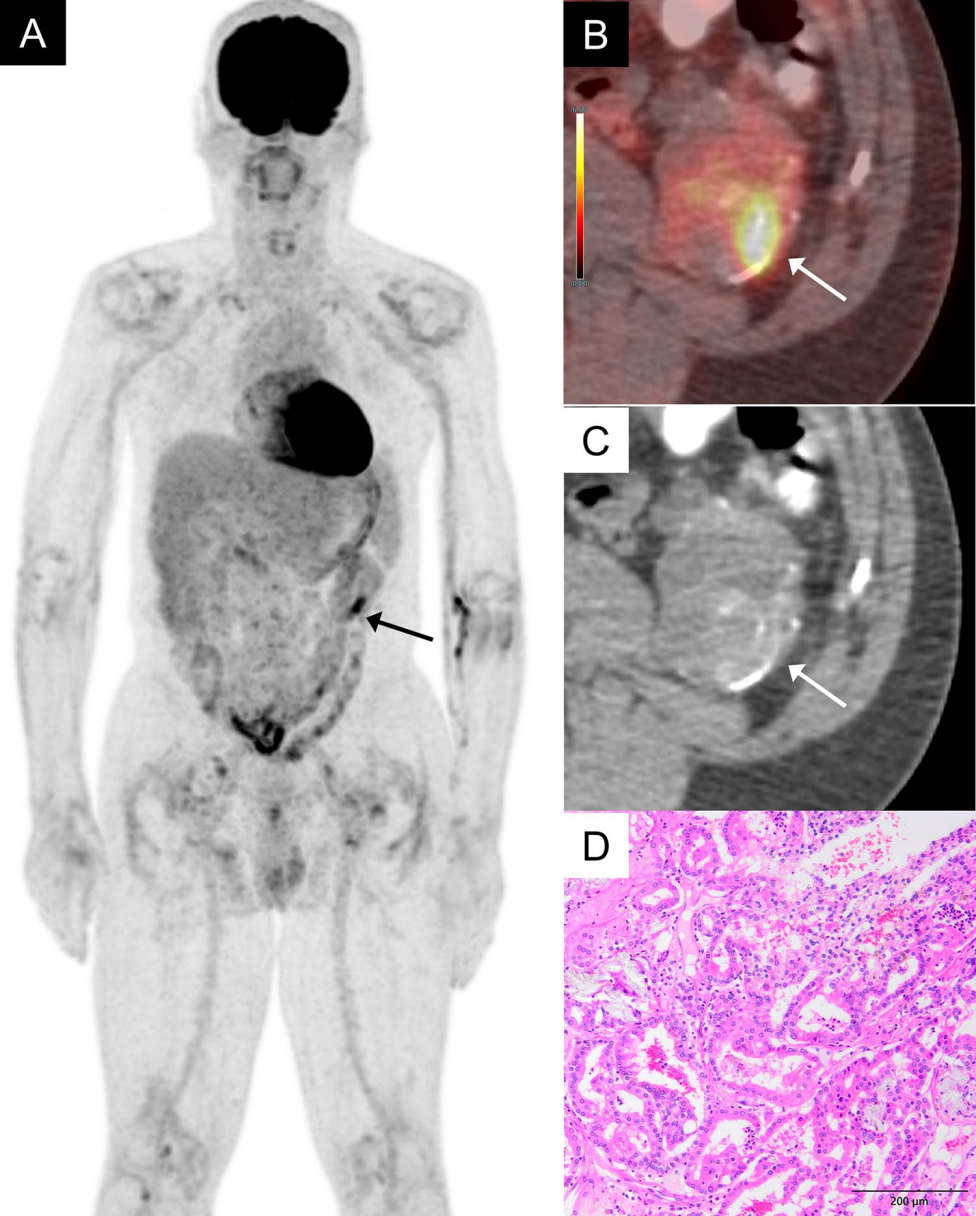
A 53-year-old man with an acquired cystic disease-associated renal cell carcinoma of the left kidney. **A** In the Maximum Intensity Projection (MIP) image of whole body, no FDG excretion into the renal urinary tract is observed, while localized accumulation (arrow) is noted in the left kidney. **B** Axial FDG-PET/CT image shows moderate FDG uptake in a mass of the left kidney (arrow), showing an SUVmax 6.9, while normal renal cortex shows an SUVmax 2.2. Notably, reduced kidney function leads to less FDG excretion through the kidney, which facilitates the detection of tumor. **C** It is difficult to identify the tumor on the CT scan. **D** The tumor shows tubular structures and calcium oxalate are observed in some areas. The tumor cells have distinct and eosinophilic nucleoli which are visible at low magnification (× 100)

ACD-RCC arises commonly in long-term hemodialysis patients. ACD-RCC often presents diagnostic challenges due to its poor contrast enhancement on contrast-enhanced CT scans. Furthermore, in patients with impaired renal function, contrast-enhanced MRI cannot be utilized due to the adverse effects of the contrast agent, which may lead to delayed tumor detection. In patients with end-stage renal disease, where FDG excretion into the urinary tract is reduced, the contrast between FDG-avid ACD-RCC lesions and the normal renal or urinary systems may be more pronounced, potentially aiding in diagnosis (Fig. 7). One study involving 150 hemodialysis patients reported that FDG-PET showed high sensitivity, specificity, and high negative predictive value (100%, 93.9%, 100%, respectively) for detecting renal cell carcinoma including ACD-RCC [8].

#### Mucinous tubular and spindle cell carcinoma (MTSCC)

**Fig. 8:**
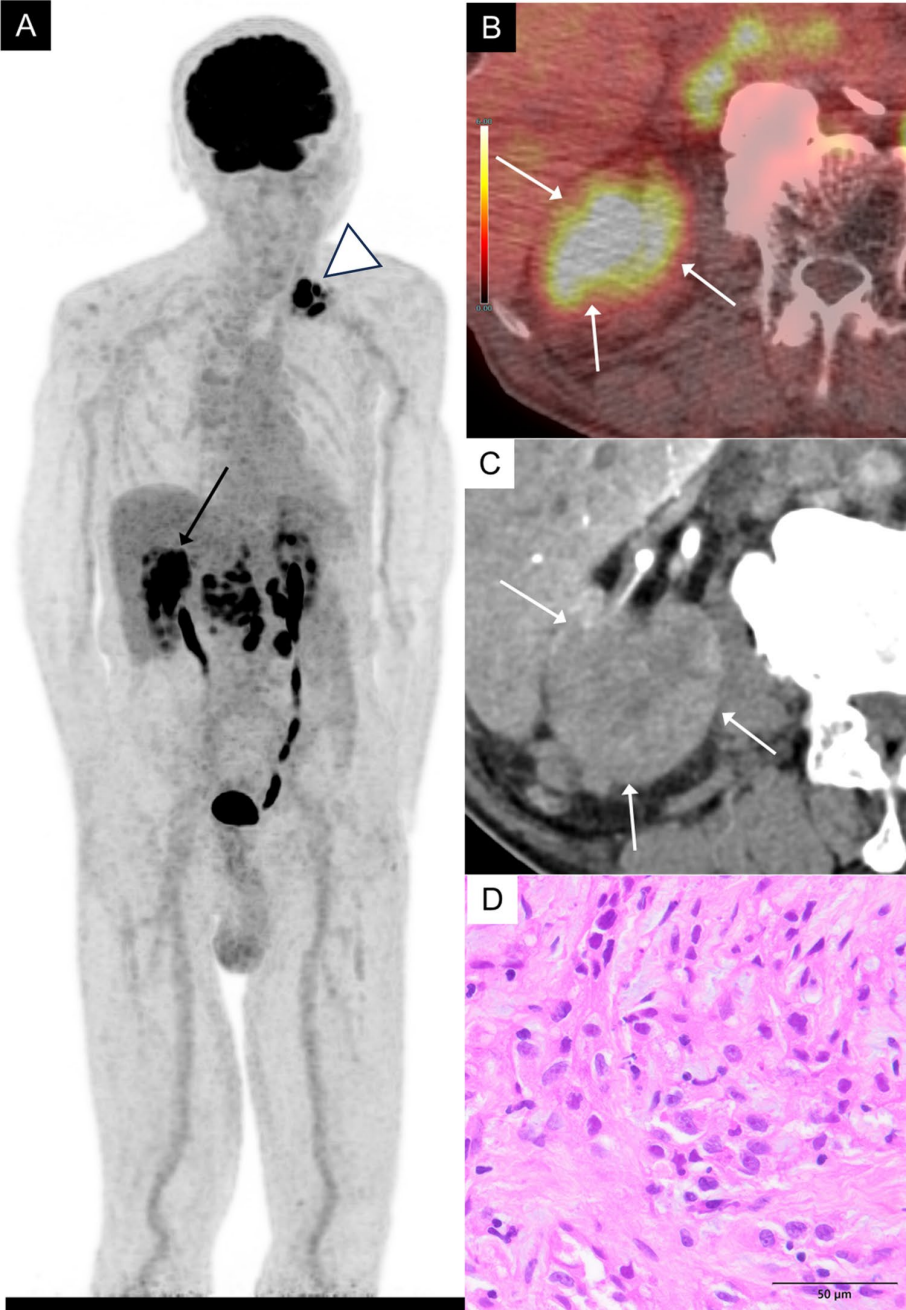
An 85-year-old man with a mucinous tubular and spindle cell carcinoma of the right kidney. **A** MIP of FDG-PET image shows intense mass-like FDG uptake in right kidney (arrow) and in the left subclavian lymph nodes (arrowhead). **B** Axial FDG-PET/CT image shows intense FDG uptake in a mass of the right kidney (arrows), showing an SUVmax 14.4, while normal renal cortex shows an SUVmax 2.7. **C** Axial contrast-enhanced CT in the corticomedullary phase shows a poor enhancement in the mass (arrows). **D** The tumor exhibits trabecular patterns with mucinous stroma. In this case, the tumor cells are more atypical than in usual cases

MTSCC is a rare renal tumor characterized by abundant mucin production. MTSCC predominantly occurs in female patients across a wide age range. It is a hypovascular renal tumor, distinct from ccRCC; however, distinguishing MTSCC from other hypovascular RCC subtypes remains challenging due to the overlap in their imaging characteristics [9]. Though few in number, reports have described elevated FDG uptake in these tumor, with SUVmax values ranging from 6.8 to 28 [10]. In our own case, FDG uptake was also elevated, with an SUVmax of 14.4 (Fig. 8).

#### Molecularly defined renal carcinomas

Several molecularly defined renal carcinomas have also been reported in the context of FDG-PET findings.

**Fig. 9:**
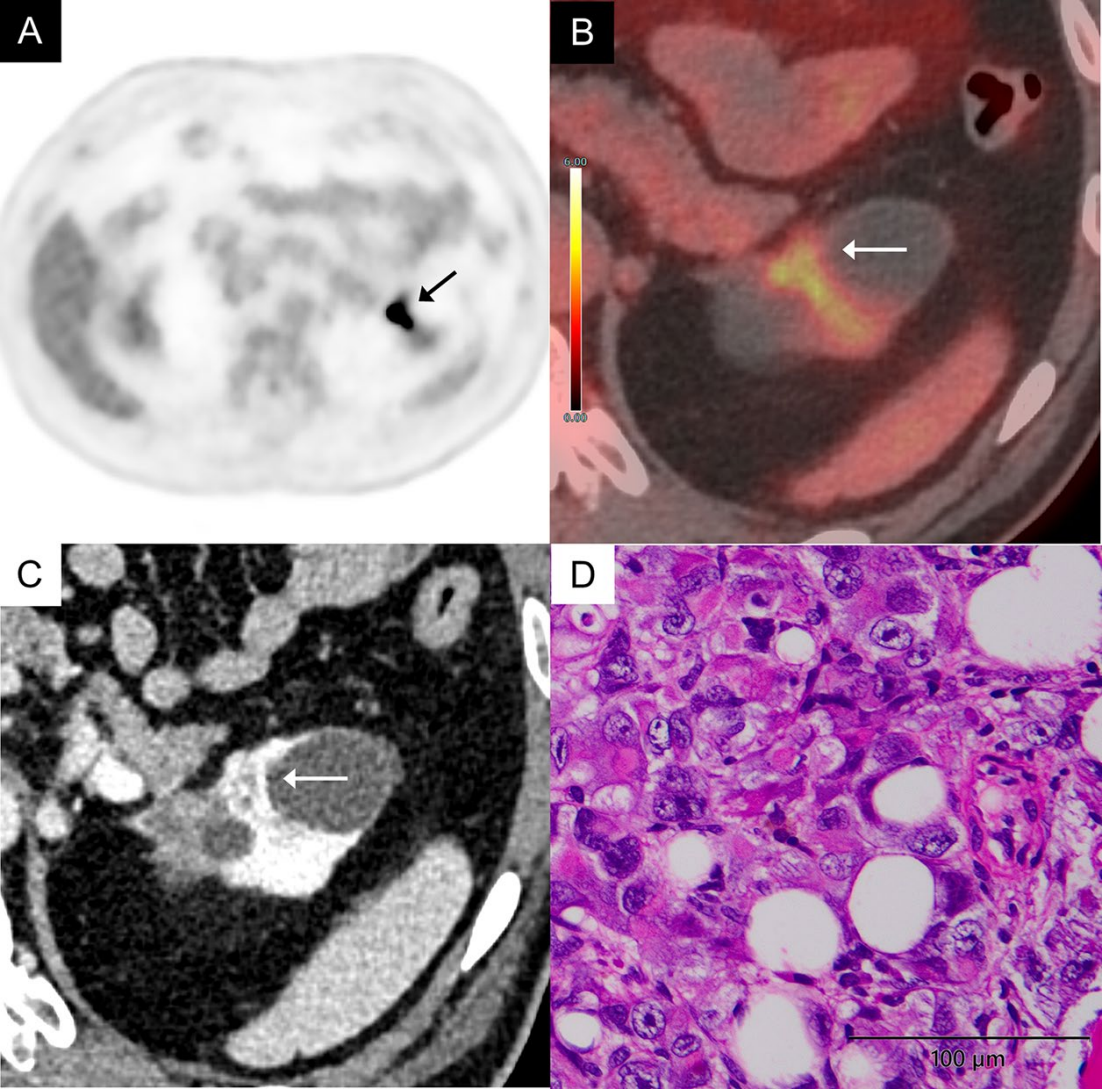
A 54-year-old man with a fumarate hydratase-deficient renal cell carcinoma of the left kidney. The patient had a history of hereditary leiomyomatosis and renal cell cancer syndrome. **A** Axial FDG-PET image shows moderate mass-like FDG uptake in the left kidney. **B** Axial FDG-PET/CT image shows moderate FDG uptake in a mass of the left kidney (arrow), showing an SUVmax 7.25, while normal renal cortex shows an SUVmax 2.8. **C** Axial contrast-enhanced CT in the corticomedullary phase shows an enhancement in the mass (arrow). Multiple osteolytic lesion are detected in the thoracic vertebra, ribs and pelvis (images not shown). **D** The biopsy of the osteolytic lesion in the thoracic vertebra shows the tumor cells which have distinct nucleoli with eosinophilic cytoplasm

- **TFE3-rearranged RCC**, which arises from fusions between the TFE3 gene and various other genes on different chromosomes, commonly occurs in children and young adults. Due to the scarcity of comprehensive reports on this disease, it is difficult to distinguish between this rare tumor and other renal tumors, such as ccRCC, papRCC and malignant lymphoma. Moderate FDG uptake which is comparable to that of high-grade RCC has been reported, with SUVmax values approximately 6 to 7 [11].
- **Fumarate hydratase-deficient renal cell carcinoma (FH-RCC)** is a very rare subtype of RCC. It may occur as part of hereditary leiomyomatosis and RCC syndrome, caused by a germline mutation, or it may arise sporadically due to a somatic mutation. Unlike conventional RCC, FH-RCC primarily affects younger individuals, mostly in their forties, and has a poorer prognosis, as it tends to metastasize early, even when the tumor is small [12]. In cases where the primary lesion is small and metastatic lesions are detected first, FDG-PET may be used to search for the primary site. These tumors exhibit high FDG uptake, with reported SUVmax values ranging from 9.6 to 21.6 [12]. In our own case, an elevated FDG uptake with an SUVmax of 7.25 was observed (Fig. 9).

### Staging and postoperative management of RCC

FDG-PET is valuable for detecting metastases in RCC, as metastatic sites often show high uptake even when primary renal tumors do not. It excels in identifying tumor thrombi, seeding, lymph node, and distant metastases, particularly osteolytic bone metastases, where the sensitivity of bone scintigraphy is limited [13]. FDG-PET is also effective in detecting postoperative recurrences, identifying bone metastases, small peritoneal seeding, or lymph node metastases that are difficult to detect with conventional CT [14]. When metastases are detected prior to the identification of the primary tumor, FDG-PET is sometimes performed to search for the primary site. Although physiological FDG uptake is observed in the kidneys, careful attention should be paid to the potential presence of renal tumors.

### FDG uptake in angiomyolipoma (AML) and RCC

**Fig. 10:**
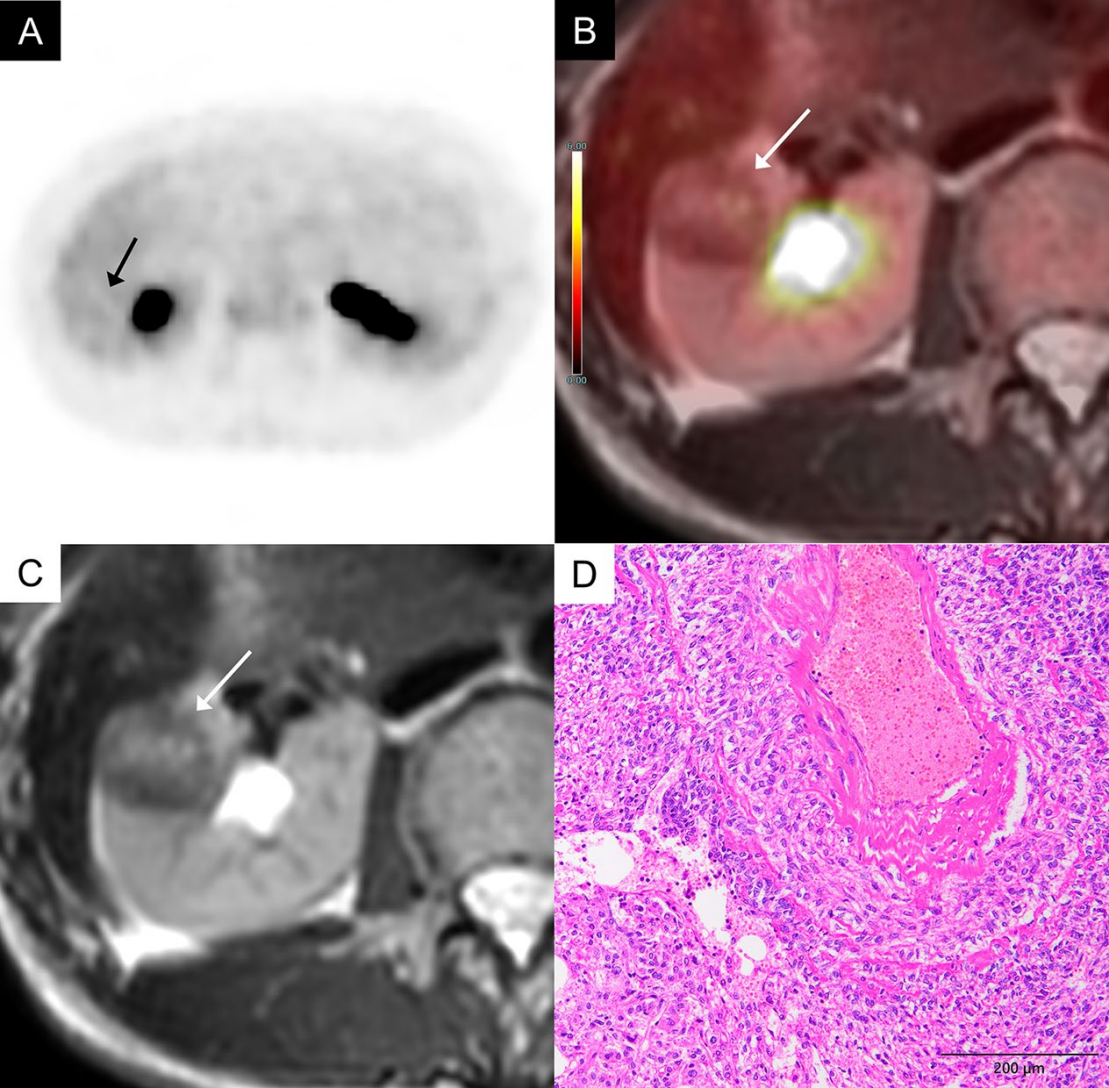
A 44-year-old woman with an angiomyolipoma of the right kidney. **A** In the FDG-PET image (grayscale), the uptake of the right renal tumor (arrow) is almost the same as that of the renal cortex, making tumor identification difficult. **B** Axial FDG-PET/MRI image shows FDG uptake in the right renal mass (arrow, SUV max: 2.49) comparable to that of normal renal cortex (SUV max: 2.08). **C** Axial T2-weighted image shows a low signal intensity in the mass (arrow). **D** The tumor consists of three components: smooth muscle cells, adipose tissue and vascular tissue. The smooth muscle cells have short spindle-shaped nuclei without high-grade morphology

AML is classified as a renal mesenchymal tumor and is the most common benign tumor of the kidney, generally exhibiting low FDG uptake (Fig. 10). An FDG-PET study of 21 AML cases showed SUV max values below 1.98, lower than those typically reported in RCC [15]. However, it should be noted that overlap may also occur. For diagnostically challenging fat-poor AML, comprehensive FDG-PET reports are lacking, though some cases show increased uptake, warranting caution [16].

## Discussion

FDG-PET is generally considered less useful for evaluating renal masses due to the physiological excretion of FDG, which reduces the contrast between normal tissue and renal tumors. The European Society for Medical Oncology (ESMO) guidelines do not routinely recommend FDG-PET for RCC [17]. However, as demonstrated in this study and previous reports, FDG-PET exhibits high efficacy in the detection of tumors in cases where FDG excretion into the renal urinary tract is limited due to chronic kidney failure. It has been reported that FDG-PET is useful in assessing the malignancy of ccRCC and papRCC [2–6], as well as in detecting metastases and recurrences of RCC [13, 14]. Furthermore, when incidental renal lesions are identified on FDG-PET, an understanding of the varying degrees of FDG uptake among different tumor types may aid in recommending appropriate subsequent diagnostic evaluations. In addition, the degree of FDG uptake in renal tumors observed on FDG-PET may serve as a potential indicator for guiding further investigations, such as genetic analysis.

Recent advancements in imaging diagnostics for RCC using FDG-PET have been made through radiomics, a technique that extracts imaging features from large volumes of radiological data using artificial intelligence. For instance, radiomics has been reported to be useful in differentiating between malignant lymphoma and RCC [18], and in predicting WHO/ISUP grades for ccRCC [19].

Furthermore, molecular imaging techniques other than FDG, such as prostate specific membrane antigen (PSMA), sestamibi, fibroblast activation protein (FAPI) and girentuximab, have shown promise in renal tumor imaging, while none of these are covered by insurance in Japan [20]. PSMA-PET may be superior to FDG-PET in detecting primary and metastatic lesions, while sestamibi might be useful in differentiating oncocytoma and chRCC from other renal tumors.

It should be noted that in the cases presented in this article and the cited references, different PET imaging devices were used; therefore, the SUV values provided should be considered as reference values.

## Conclusion

FDG-PET is a valuable tool for detecting metastases and postoperative recurrence of RCC and is also useful in the evaluation of renal tumors. Although physiological FDG uptake occurs in the kidneys, meticulous attention should be given to identifying renal lesions. While the utility of dynamic CT and MRI in the qualitative diagnosis of renal tumors is well established, a deeper understanding of FDG uptake patterns in different renal tumor subtypes may allow FDG-PET to serve as a useful adjunctive imaging modality. In the future, advancements in radiomics and novel molecular imaging techniques hold the potential to further enhance the accuracy and effectiveness of renal tumor imaging.
